# Differential responses of myoblasts and myotubes to photobiomodulation are associated with mitochondrial number

**DOI:** 10.1002/jbio.201800411

**Published:** 2019-02-20

**Authors:** Hannah J. Serrage, Sophie Joanisse, Paul R. Cooper, William Palin, Mohammed Hadis, Owen Darch, Andrew Philp, Mike R. Milward

**Affiliations:** ^1^ School of Dentistry College of Medical and Dental Sciences, Institute of Clinical Sciences, University of Birmingham Birmingham UK; ^2^ School of Sport, Exercise and Rehabilitation Sciences University of Birmingham Birmingham UK; ^3^ Philips Research Eindhoven The Netherlands; ^4^ Garvan Institute of Medical Research Darlinghurst New South Wales Australia

**Keywords:** low‐level laser therapy, low‐level light therapy, mitochondria, myogenesis, photobiomodulation

## Abstract

**Objective:**

Photobiomodulation (PBM) is the application of light to promote tissue healing. Current indications suggest PBM induces its beneficial effects in vivo through upregulation of mitochondrial activity. However, how mitochondrial content influences such PBM responses have yet to be evaluated. Hence, the current study assessed the biological response of cells to PBM with varying mitochondrial contents.

**Methods:**

DNA was isolated from myoblasts and myotubes (differentiated myoblasts), and mitochondrial DNA (mtDNA) was amplified and quantified using a microplate assay. Cells were seeded in 96‐wellplates, incubated overnight and subsequently irradiated using a light‐emitting diode array (400, 450, 525, 660, 740, 810, 830 and white light, 24 mW/cm^2^, 30‐240 seconds, 0.72‐5.76J/cm^2^). The effects of PBM on markers of mitochondrial activity including reactive‐oxygen‐species and real‐time mitochondrial respiration (Seahorse XFe96) assays were assessed 8 hours post‐irradiation. Datasets were analysed using general linear model followed by one‐way analysis of variance (and post hoc‐Tukey tests); *P* = 0.05).

**Results:**

Myotubes exhibited mtDNA levels 86% greater than myoblasts (*P* < 0.001). Irradiation of myotubes at 400, 450 or 810 nm induced 53%, 29% and 47% increases (relative to non‐irradiated control) in maximal respiratory rates, respectively (*P* < 0.001). Conversely, irradiation of myoblasts at 400 or 450 nm had no significant effect on maximal respiratory rates.

**Conclusion:**

This study suggests that mitochondrial content may influence cellular responses to PBM and as such explain the variability of PBM responses seen in the literature.

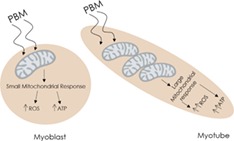

## Graphical abstract

We demonstrate novel findings that suggest the effects of photobiomodulation (PBM) are mitochondrial content dependent. In which muscle cells with different mitochondrial contents were evaluated for their response to PBM. It was found that cells possessing a higher mitochondrial content (myotubes) exhibited a greater real‐time mitochondrial response to, in particular, blue light (400‐450 nm).

## 1 INTRODUCTION

Photobiomodulation (PBM) is a non‐invasive treatment that utilises light at a power output less than 500 mW at wavelengths from 400 to 1100 nm to promote tissue healing, reduce inflammation and induce analgesia 1. Over 800 publications have reported the efficacy of PBM in treating an array of musculoskeletal conditions including subacute and chronic low back pain 2 and exercise induced muscle fatigue 3. Musculoskeletal disorders relate to an array of conditions that affect movement and are a significant burden, not only to the affected individual but also to healthcare systems due to the costs associated with management. Indeed, a report by the World Health Organization concluded that up to 33% of the population are affected by lower back pain at any given time 4.

Despite the positive evidence surrounding the use of PBM in treating musculoskeletal conditions, controversy still surrounds its application in practice due to a lack of consistency in the recording of treatment parameters. Notably, a number of key irradiation parameters should be reported including wavelength (nm) and irradiance (mW/cm^2^), amongst others 5. These parameters are often either misreported or not reported at all making it difficult to compare literature currently published.

Another key caveat in the use of PBM is the lack of knowledge as to how light energy elicits its beneficial molecular effects. Current literature indicates light acts directly upon the mitochondrial electron transport chain (ETC), specifically complex IV 6. The ETC is formed of five complexes, and its main purpose is to produce adenosine triphosphate (ATP), the cells energy source. It is understood that photons of light at wavelengths including 810 nm excite complex IV, causing the dissociation of nitric oxide (NO) from its binding site, allowing oxygen to bind in its place and therefore allowing the progression of the ETC 6, 7. As the ETC progresses, complexes I and III of the chain also produce reactive oxygen species (ROS). The production of ROS and ATP then induce the activation of transcription factors and subsequent gene expression changes including increased nuclear factor E2‐related factor 2 (Nrf2) 8, a gene whose expression is commonly associated with increased mitochondrial biogenesis 9.

Many studies report the effect of PBM on mitochondrial activity through the use of surrogate assays including ROS 10 and ATP 11 generation. However, despite wide evidence supporting this ideology, no studies to date report the effects of PBM on mitochondrial respiration or whether mitochondrial number can influence response to PBM. In fact, the number of mitochondria per cell can vary from 80 to 2000 dependent upon the cell type explored 12. Robin and Wong reported that there are approximately 1000 mitochondria per liver cell, whilst there are around 300 mitochondria per human lung fibroblast cell 13.

This study aimed to first characterise a system that could be employed to evaluate the effects of PBM on changes in mitochondrial respiration in real time and second to determine the optimal treatment parameters that elicit a molecular response in muscle‐derived cells in which mitochondrial activity is key to their behaviour. C2C12 myoblasts have been suggested to be an appropriate model for mimicking the process of skeletal muscle cell differentiation in vitro 14. When exposed to appropriate conditions myoblasts differentiate into myotubes in vitro 15. Mature myotubes are cited to have a higher population of mitochondria than myoblasts 16, 17. Hence, myoblasts and myotubes were employed to determine whether cells with a higher mitochondrial population responded differently to PBM.

## 2 METHODS

### 2.1 Light‐emitting diode array characterisation

#### 2.1.1 Spectral characterisation

A UV–Vis spectrometer (USB4000; Ocean Optics, Largo, Florida) coupled to a 200‐μm optical fibre and 3.9‐mm cosine corrector and calibrated to the National Institute of Standards and Technology (NIST) standards was employed to assess the spectral irradiance and wavelength delivered at the base of each individual culture well (n = 6). Absolute irradiance was determined from the integral of the spectral irradiance (380‐880 nm). Further detail outlining spectral characterisation methods, light‐emitting diode (LED) array design and selection of wavelengths are described by Hadis et al 18.

#### 2.1.2 Beam profile

A charge coupled device beam profile camera (SP620; Ophir, Spiricon, Israel) was employed to measure spatial distribution of power emitted from each LED in the array. A 50‐mm closed‐circuit television lens (Ophir) was attached to the camera and focused on the base of each well. Following linear, optical and ambient light correction, images were recorded using BeamGage software (Ophir). Detailed experimental procedure has previously been reported by Hadis et al 18.

### 2.2 Biological responses

#### 2.2.1 Myoblasts culture

A mouse myoblast cell line (C2C12 [American Type Culture Collection (ATCC) CRL‐1722], ATCC, LGC standards, UK, passage 8‐11) 14 was cultured in monolayers in Dulbecco's modified eagle medium (DMEM, Gibco, Thermo‐Fischer Scientific, Waltham, Massachusetts) supplemented with 10% v/v fetal calf serum, 1% v/v penicillin/streptomycin (P/S) and 1% v/v l‐glutamine (Sigma‐Aldrich, St Louis, Missouri). Cells were seeded into 96‐well black clear bottom plates (7000 cells/well; Sigma‐Aldrich) and Seahorse XFe96 plates (10,000 cells/well; Agilent, Santa Clara, California), incubated overnight (37°C, 5% CO_2_>), irradiated as described in Section 2.2.3 and changes in mitochondrial activity were assessed 8 or 24 hours post‐irradiation.

#### 2.2.2 Myotube differentiation

Myoblast cultures were seeded into Seahorse XFe96 plates as described in Section 2.2.1 and approximately 70% confluency was reached, cultures were washed with phosphate buffered saline (PBS) and differentiation media was subsequently applied which contained phenol red free DMEM containing 2% v/v horse serum and 1% v/v sodium pyruvate (Sigma‐Aldrich) to induce differentiation for 6 days. Myotubes were then irradiated as described above, and changes in mitochondrial activity were assessed 8 hours post‐irradiation.

#### 2.2.3 Array characterisation for Seahorse XF cell mitochondrial stress assay [Agilent]

A Seahorse XFe96 Analyser (Agilent) was employed to measure the cells’ oxygen consumption rate (OCR) as a marker of mitochondrial respiration. One hour prior to undertaking the assay, culture media was aspirated, cells washed with PBS three times and Seahorse XF assay media (25‐mM glucose, 1‐mM pyruvate and 2‐mM glutamine; Agilent) was applied and equilibrated in a CO_2_ free incubator (INCU‐line; VWR, Radnor, Pennsylvania). Compounds altering mitochondrial activity were then applied to the system including: Oligomycin (inhibits complex V of the ETC, 1 μM), carbonyl cyanide‐4(triflouromethoxy)phenylhydrazone (FCCP, uncoupling agent induces respiration to be undergone at maximal rates, 2 μM), antimycin and rotenone A (inhibit complexes I and III, inhibiting ETC activity, 0.5 μM). Subsequently, the plate was placed in a Seahorse XFe96 analyser (Agilent) and compounds were sequentially injected into the system to induce changes in ETC activity. The Seahorse analyser then measured changes via assessment of OCR in real time (OCR, pmol/min). OCR values were subsequently normalised for protein content in individual wells. Protein concentration was determined using detergent compatible protein assay (Bio‐rad, Hercules, California). This enabled calculation of individual parameters including basal respiration, maximal respiration, ATP production, spare respiratory capacity and non‐mitochondrial respiration 8. During analysis, values for non‐mitochondrial activity were subtracted from values evaluating the effects of PBM directly on the mitochondrial activity. This provided a further control step ensuring results would reflect the effects of PBM on mitochondrial activity only.

**Figure 1:**
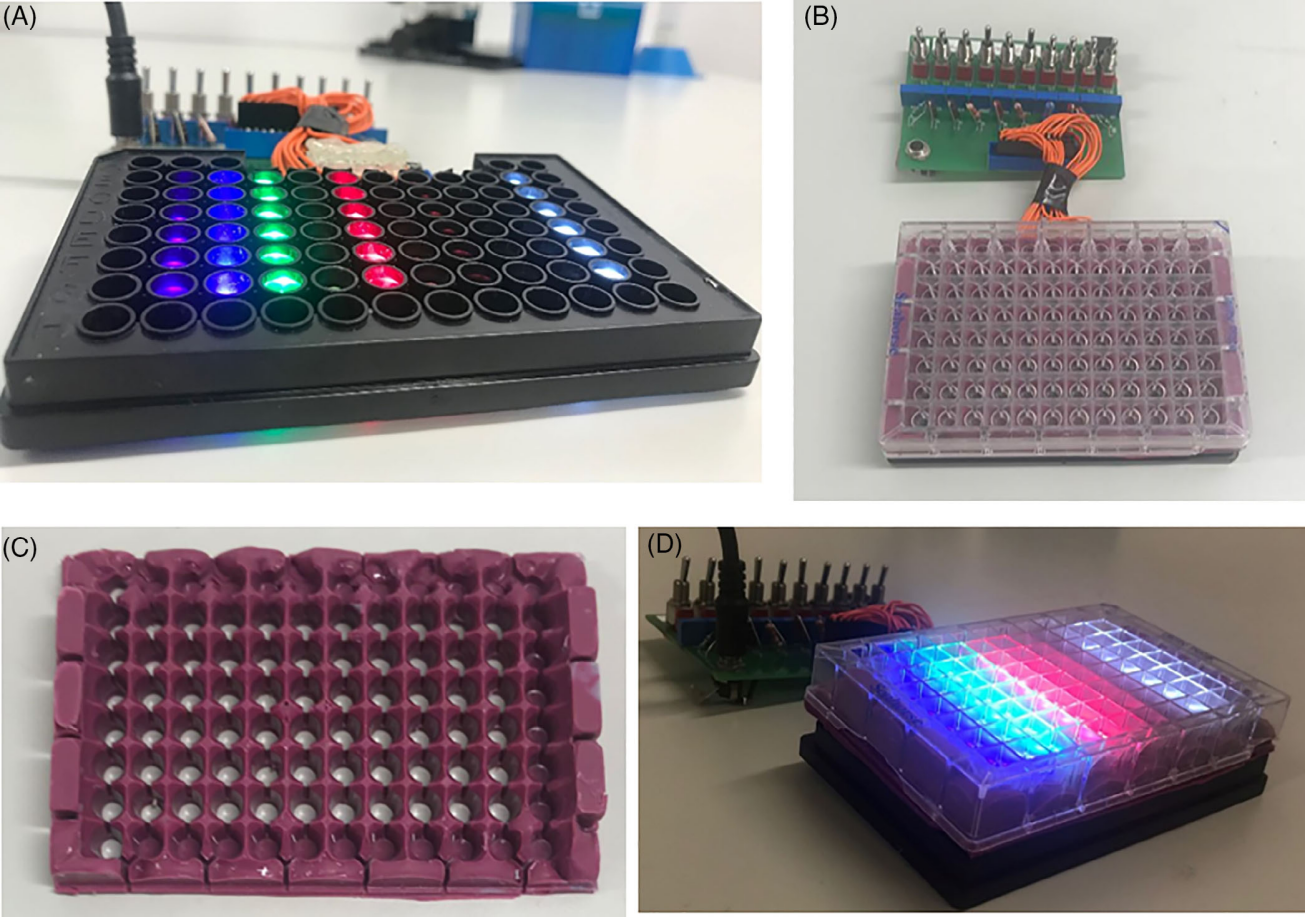
Indicates design and experimental setup of the LED array in 96 well format in which shows (A) LED array without plate‐overlaid (B) array overlaid with 96 well black clear bottom plate (no wavelengths on) (C) mask constructed from opaque dental silicone impression material and (D) seahorse XFe96 plate fitted with mask and placed upon LED array when on (all wavelengths). All spectral characterisation experiments were undertaken with the Seahorse plate fitted mask placed directly above the LED array ensuring concentric alignment with LEDs beneath

An opaque dental silicone impression material (3 M; Impregum Penta Soft, Maplewood, Minnesota) mask (Figure 1B) was created to ensure uniform irradiation of in vitro cultures and to eliminate light bleed at the base of wells where cells adhere (Figure 1C and D). The distance between the LEDs and specimen surface was fixed at 3 mm in each well. The spectral irradiance and beam profile of each diode were evaluated with the mask fitted to a Seahorse microplate (Figure 1D). Characterisation was then undergone as described in Section 2.1. The effects of PBM were evaluated at wavelengths spanning the visible and near infrared spectra (400‐830 nm) at irradiation periods between 30 and 240 seconds and an irradiance output of 24 mW/cm^2^ to achieve radiant exposures of 0.72 to 5.76 J/cm^2^.

#### 2.2.4 Mitochondrial DNA quantification

A REPLI‐g mitochondrial DNA kit (Qiagen, Hilden, Germany) was used to amplify mtDNA in whole DNA samples isolated from cell cultures. Sample DNA concentrations were measured spectrophotometrically (Eppendorf Biophotometer, Eppendorf, UK) and diluted accordingly to contain 10 ng/μl of DNA. Amplification of mtDNA was then undertaken according to the manufacturer's protocol.

To assess mtDNA quantities in cell supernatants, initially a standard curve was generated using calf thymus DNA at a maximal concentration of 10 ng/μl. SYBR Safe DNA gel stain (10 000× concentrate; Invitrogen, Carlsbad, California) was diluted in Tris‐acetate‐EDTA buffer at 1:1250. Samples and standards were then combined at 2% v/v with the dilute SYBR Safe dye and incubated for 10 minutes 19. Fluorescence was measured using a fluorimeter (Twinkle LB 970, 485 nm/535 nm, excitation/emission, respectively; Berthold Industries Ltd., Bad Wildbad, Germany).

#### 2.2.5 3‐(4, 5‐dimethylthiazol‐2‐yl)‐2,5‐diphenyltetrazolium bromide assay

To assess cell metabolic activity, a 3‐(4, 5‐dimethylthiazol‐2‐yl)‐2,5‐diphenyltetrazolium bromide (MTT) assay (Sigma‐Aldrich) was utilised 20. MTT was dissolved in PBS at 0.05 g/mL and aliquoted at 15 μL/well 8/24 hours post‐irradiation and incubated for 4 hours at 37°C. MTT solution was aspirated and replaced with 50 μL/well of dimethyl sulphoxide (Sigma‐Aldrich). Absorbance was read at 570 nm using a microplate reader (ELx800 Universal Microplate reader; Bio‐Tek Instruments, Winooski, Vermont).

#### 2.2.6 ROS assay

ROS formation was assessed using 2′, 7′‐dichlorodihydrofluorescein diacetate (H_2_DCFDA) fluorescent probe (Thermo‐Fischer Scientific, Waltham, Massachusetts). Free radicals catalyse the conversion of H_2_DCFDA to its fluorescent bi‐marker 2′,7′‐dichlorofluorescein, enabling quantification of ROS production. At 8 hours post‐irradiation media was aspirated, cells were washed with PBS and were treated with 10‐μm H_2_DCFDA and incubated for 1 hour at 37°C 21. Fluorescence was read using a fluorimeter as described in Section 2.2.3.

### 2.3 Statistical analysis

Data were processed utilising Excel software (Microsoft Redmond, Washington), and analysis was performed using SigmaPlot software (Systat Software Inc, San Jose, California). All data were analysed using a general linear model followed by one‐way analysis of variance test followed by a Tukey test to determine significant differences between non‐irradiated controls and light treated groups (*P* < 0.05).

## 3 RESULTS AND DISCUSSION

### 3.1 Characterisation of LED arrays for use in seahorse assays

LED arrays provide a high‐throughput approach for analysis of multiple parameters and their effects in vitro. The current study first aimed to characterise a system that could be employed for use with the Seahorse XFe96 analyser system.

**Figure 2:**
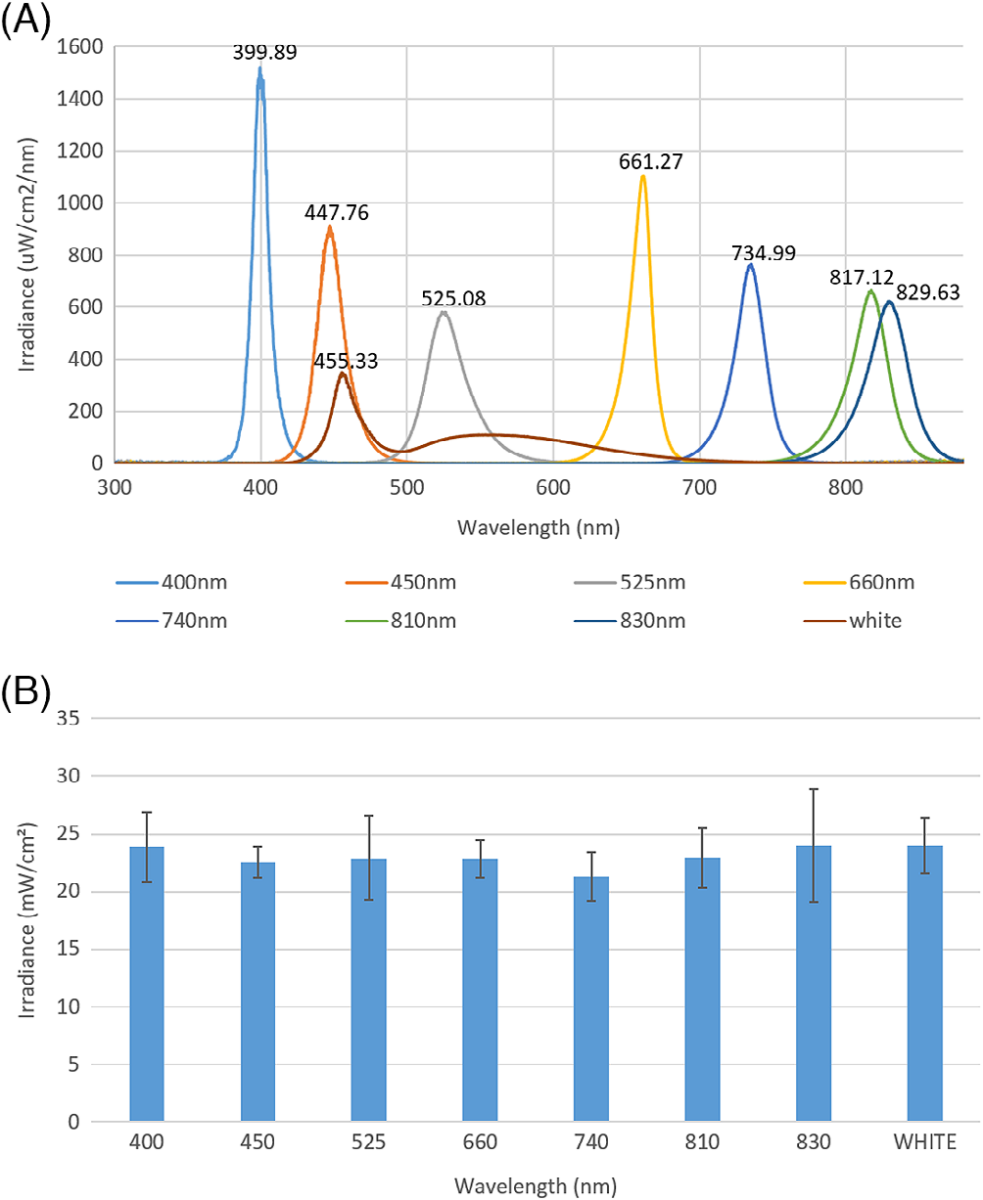
Array spectral characterisation data where (A) Spectral irradiance values of LED channels in the array (n = 6) and (B) average absolute irradiance in each channel (n = 6)

A mask constructed from silicon was designed to surround each well of the plate, preventing bleed between wells to ensure only a single wavelength of light would impact the biological response in each well‐culture. Spectral characterisation undertaken to confirm wavelength and spectral irradiance values were consistent with those used in previous studies employing different plate formats 18. Data indicated the array employed for in vitro studies emitted wavelengths ranging from 400 to 830 nm (Figure 2A) and an average irradiance of 23.05 mW/cm^2^ (Figure 2B). These data also confirmed there was no bleed of light between LED columns where Figure 2A indicates LEDs exhibited only a single peak of the expected wavelength and Figure 2B shows there was no significant difference in irradiance output from one wavelength to the next. Similar irradiance values have also been used in studies exploring the effect of PBM on myoblast function (see Table S3 in Appendix S1, for examples, 22, 23). Table 1 indicates radiant exposure values in which there is no significant difference from one wavelength to the next (irradiation parameters and the effects of PBM on media temperature are further elucidated in Figure S1, Figure 2 and Table 1). Hence, the data confirm the accurate delivery of key radiometric parameters without confounding effects such as temperature.

**Table 1:** Indicates values of emitted wavelengths, spectral irradiance and radiant exposure after irradiation periods ranging between 30 and 240 seconds

Wavelength (nm)	Radiant exposure (J/cm^2^)			
	30 seconds	60 seconds	120 seconds	240 seconds
399.89 ± 1.55	0.72	1.43	2.86	5.73
447.76 ± 1.69	0.68	1.35	2.70	5.41
525.08 ± 1.25	0.69	1.37	2.75	5.50
661.27 ± 0.56	0.68	1.37	2.74	5.48
734.99 ± 1.21	0.64	1.28	2.55	5.11
817.12 ± 0.64	0.69	1.37	2.75	5.50
829.63 ± 2.64	0.72	1.44	2.88	5.76
455.33 ± 0.11	0.72	1.44	2.87	5.75
Average	0.69 ± 0.03	1.38 ± 0.05	2.76 ± 0.10	5.53 ± 0.21

**Figure 3:**
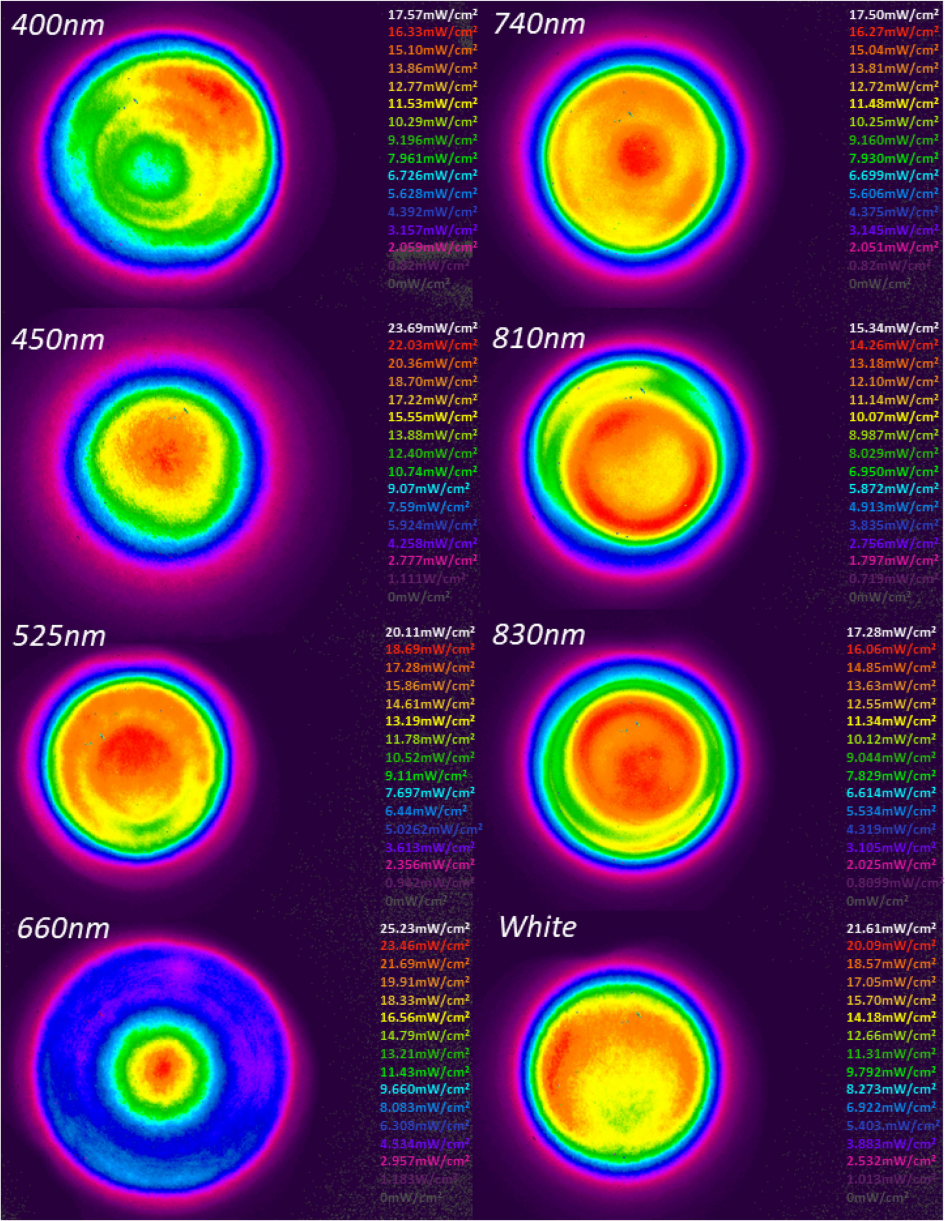
Demonstrates spatial distribution of irradiance of LEDs emitting each wavelength on the array. Images were taken in the plane of a target screen placed over the array surface to enable accurate measurement of beam diameter using BeamGage software. The target screen was placed at the same distance away from the array as a Seahorse XFe96 plate. Whilst the target screen could not be incorporated with the plate in place Figure S3 and Table S1 indicate beam profiles, average beam areas and power output with the plate in place

Another key parameter to be considered was that of the beam profiles of each LED utilised in this array. LEDs employed in this array exhibit a typical Gaussian distribution of light (Figure 3A, indicates a single representative from each wavelength channel) in which spectral irradiance is most intense in the central area and becomes more diffuse towards the edges of the beam area 24. Table 2 indicates LEDs emitting a wavelength of 525 nm exhibited a significantly smaller (*P* < 0.05) beam area and power output than LEDs emitting wavelengths of 400 and 660 nm, whilst there was no significant difference at all other wavelengths. In this particular experimental setup, the LED array was designed to enable alignment of LEDs with the plate directly above at a specific irradiance value. As described by Hadis et al 18 that whilst there is variability in the homogeneity of each LED, the effects of this have been minimised through ensuring there is no significant difference in the output of a series of parameters including irradiance (mW/cm^2^) and radiant exposure (J/cm^2^). However, despite this it will be important to take into account the effects this may have on the biological output of our experiment. These data indicate the importance of evaluation of beam area.

**Table 2:** Indicates differences in average beam area and power output emitted from one wavelength to the next

Wavelength (nm)	Average Beam area (cm^2^)	Power (mW)
400	0.370 ± 0.008^A^	8.931 ± 0.184^A^
450	0.337 ± 0.003^AB^	8.484 ± 0.063^AB^
525	0.257 ± 0.007^B^	6.039 ± 0.157^B^
660	0.364 ± 0.003^A^	9.01 ± 0.068^A^
740	0.298 ± 0.007^AB^	6.834 ± 0.159^AB^
810	0.335 ± 0.007^AB^	7.688 ± 0.158^AB^
830	0.307 ± 0.01^AB^	7.453 ± 0.233^AB^
White	0.291 ± 0.009 ^AB^	7.721 ± 0.242^AB^

Means that do not share the same letter are significantly different, in which LEDs emitting wavelengths of 400 and 660 nm (A) exhibit significantly larger beam areas and power outputs than LEDs emitting 525‐nm light (B, *P* = <0.05). Average beam area was calculated from diameters provided from the use of BeamGage software.

The data obtained for array characterisation indicate its suitability for use in subsequent in vitro assay application.

### 3.2 The effects of PBM on mitochondrial activity

**Figure 4:**
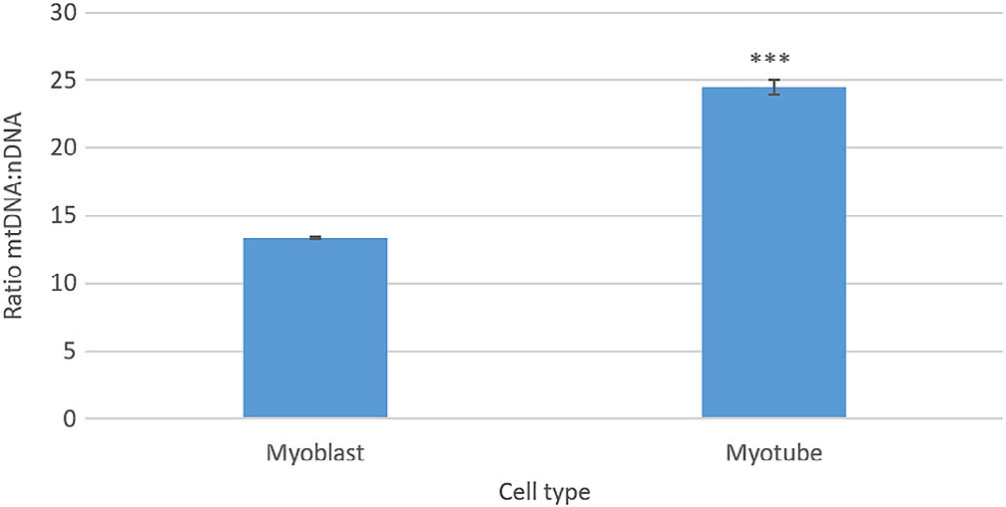
Shows relative differences in the ratio of mtDNA:nDNA between myoblasts and myotubes (n = 4, p8). Significance was assessed using a *t* test (*** = *P* < 0.001)

**Figure 5:**
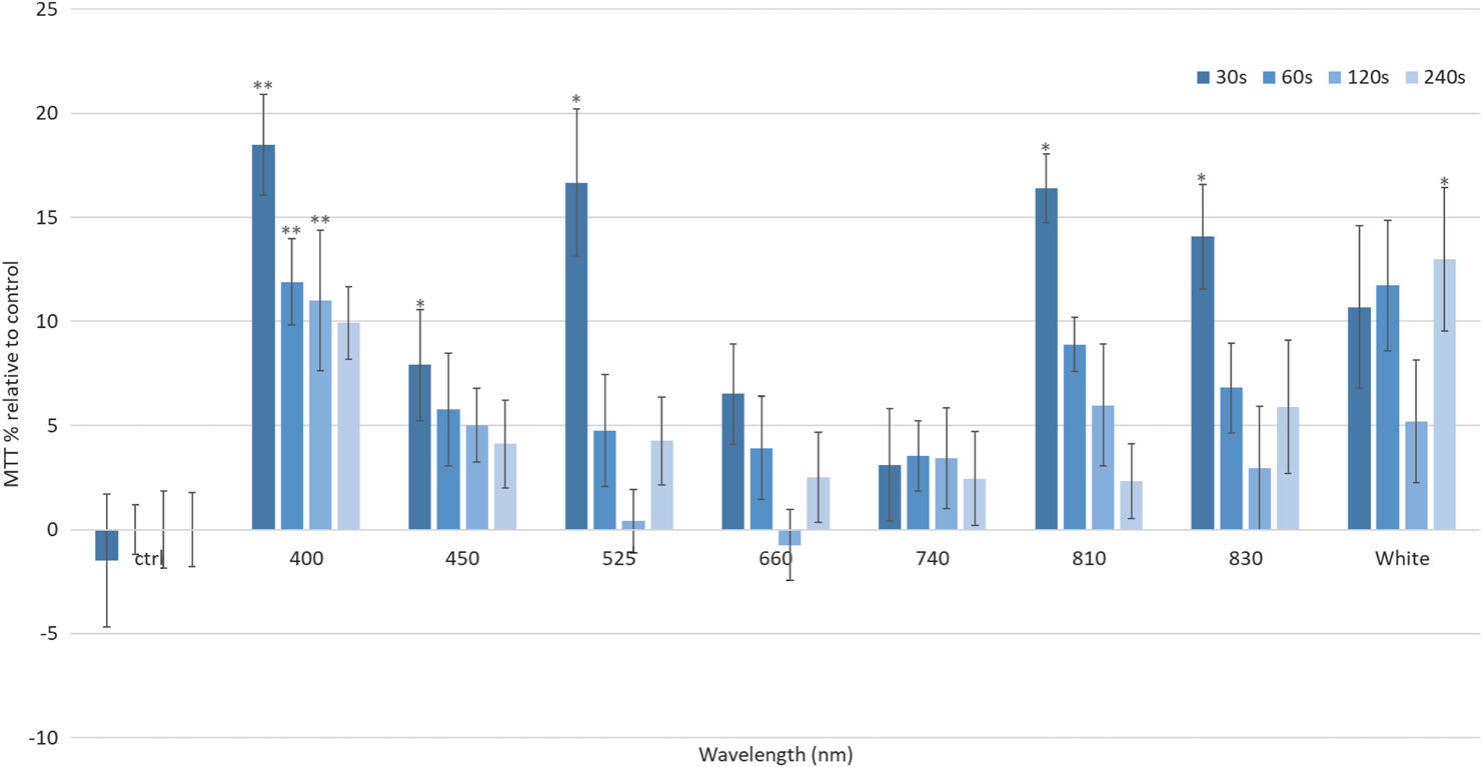
Indicates high‐throughput analysis of wavelengths (400‐830 nm) and irradiation periods (30‐240 seconds) on cell metabolic activity of mouse myoblast cells (C2C12, n = 12 replicates, three plates irradiated) (24 mW/cm^2^, 0.72‐5.76J/cm^2^, 30‐240 seconds). Significance is indicated by ** = *P* < 0.01, * = *P* < 0.05 relative to the non‐irradiated control, where all data are shown as a percentage of the non‐irradiated control, where the non‐irradiated control was normalised to 0%

The second objective of this study was to evaluate the effects of PBM on mitochondrial activity of myoblasts and myotubes. Myotubes are reported to possess higher quantities of mitochondrial proteins and enzymes 25 and hence greater numbers of mitochondria. Whilst it is not feasible to directly measure the number of mitochondria per cell due to the dynamic nature of mitochondria, mtDNA copy number has been correlated to mitochondrial content in previous studies 26. Hence, mtDNA was isolated from both myoblasts and myotubes and quantified. Figure 4 provides evidence that myotubes possessed a greater ratio of mtDNA:nDNA compared with myoblasts (*P* < 0.05). Therefore, an MTT assay was employed as a high‐throughput method to examine the effects of a series of wavelengths (400‐830 nm) and irradiation times (30‐240 seconds) on the cell metabolic activity of myoblasts 24 hours post‐irradiation. Irradiation for 30 seconds at wavelengths of 400 and 810 nm induced 18.46%, and 16.38% increases in cell proliferation, respectively (Figure 5, *P* < 0.05). Interestingly, white light induced a significant increase in cell proliferative capacity following irradiation for 240 seconds, whilst all other wavelengths proved to induce the greatest affect following a 30‐second irradiation period. This may be reflective of the differential biphasic dose response from one wavelength to the next where longer or shorter periods of irradiation could cause the most significant effects dependent upon the wavelength used. Also, white light is a combination of multiple visible light wavelengths and therefore the contribution of a single wavelength for any potential therapeutic effect within the white light band must be substantially reduced compared to the use of narrower wavebands at similar irradiance. It can also be noted that whilst wavelengths within the red spectra are commonly used in PBM research (620‐750 nm) 27, no effect on cell metabolic activity was measured here. This may be due to the homogeneity of the beam profile at 660 nm (Figure 3) or indeed as discussed above, the optimal range for red light to induce an effect was not reached in this experimental setup. Hence, further study will be required to determine whether irradiation at 660 nm and with a more homogenous beam profile will influence biological output in vitro. Whilst other wavelengths and irradiation periods proved effective in inducing a significant response from myoblasts, wavelengths of 400, 450 and 810 nm and an irradiation period of 30 seconds were selected to compare the response of myoblasts and myotubes to PBM due to their efficacy in inducing a response from an array of other cell types previously studied (data not shown).

**Figure 6:**
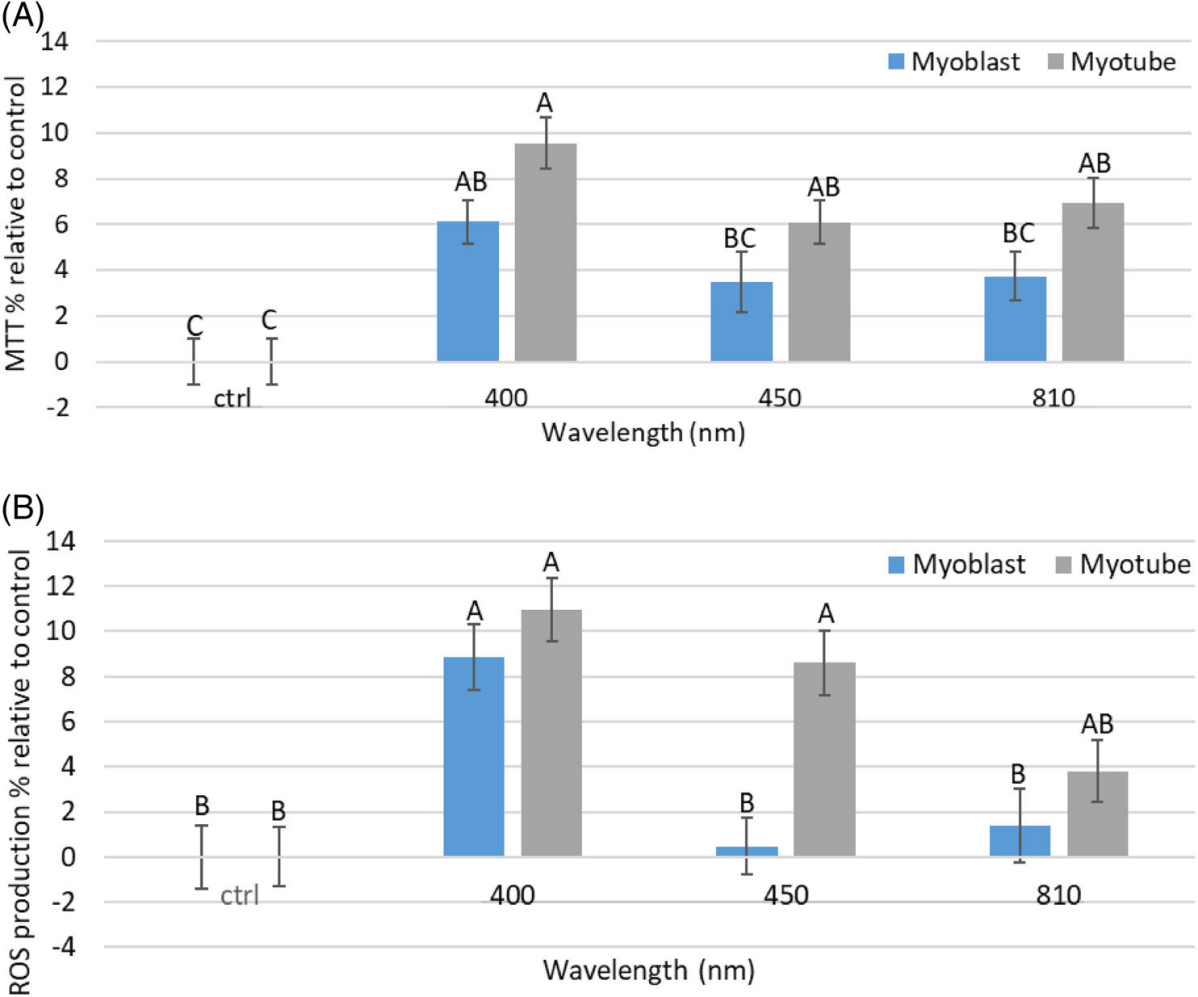
(A) Effect of PBM on cell metabolic activity from myoblasts and myotubes (30 seconds, 0.72 J/cm^2^, n = 18, three plates irradiated) to wavelengths of 400, 450 and 810 nm. (B) Indicates the effects of PBM on ROS production from myoblasts and myotubes (30 seconds, 0.72 J/cm^2^, n = 18, three plates irradiated). The effects of PBM were evaluated 8 hours post‐irradiation. Means that do not share the same letter are significantly different (*P* < 0.05)

Once parameters were identified for further study, a series of key markers for mitochondrial activity were explored 8 hours post‐irradiation. A period of 8 hours post‐irradiation was selected as a previous study indicated this time point post‐irradiation induced the most significant and reliable changes in real‐time mitochondrial activity (Figure S4, [1, 8 and 24 hours post‐irradiation were evaluated]). Figure 6 indicates that whilst a wavelength of 400 nm and irradiation period of 30 seconds induced significant increases in markers for mitochondrial activity from myoblasts and myotubes, increases in the activities of these mitochondrial markers at all wavelengths were only observed in myotube cultures (*P* < 0.05). This may indicate that cells with higher mitochondrial content have increased responsivity to light. Interestingly, Kushibiki et al investigated the effect of PBM at wavelengths of 405 and 808 nm at 100 mW/cm^2^ on ROS production from C2C12 cells. They found that only violet‐blue light upregulated ROS production whilst near infrared light had no effect. Our data provide similar findings, with a wavelength of 400 nm inducing significant increases in ROS production from myoblasts 28. Some authors have also reported the effects of PBM in inducing myogenic differentiation from myoblasts to myotubes 29, 30. Hence, future work may involve evaluation of the effects of parameters illustrated in this study on markers for myogenic differentiation.

**Figure 7:**
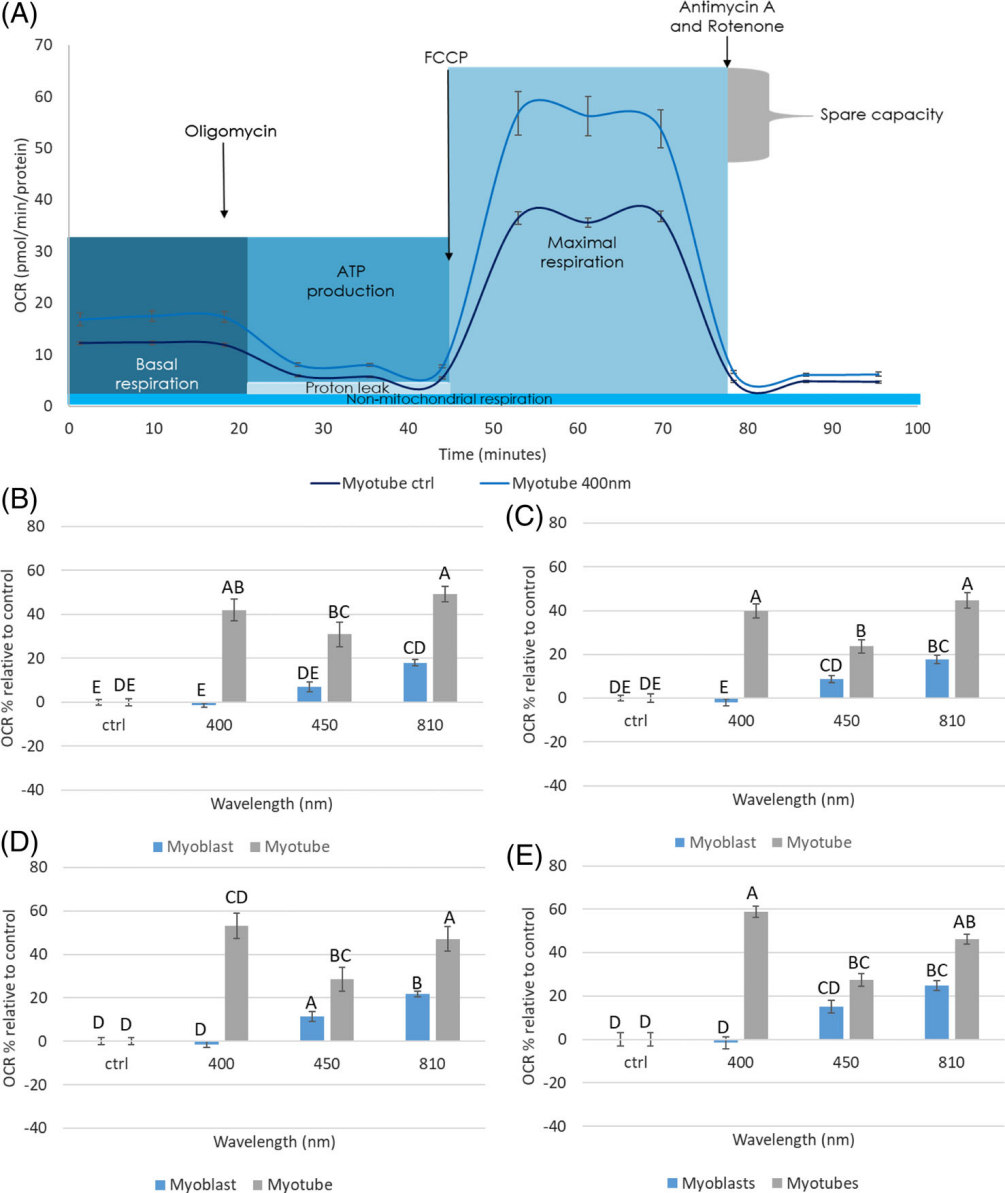
Shows the effects of PBM on markers for changes in real‐time mitochondrial activity utilising the Seahorse mitochondrial stress assay from myoblasts and myotubes (myotubes, p7, myoblasts p13, n = 6, effects evaluated 8 hours post‐irradiation). Markers are denoted by (A) indicates trace comparing response of untreated myotubes and myotubes treated with 400‐nm light in which compounds were sequentially applied to the system to alter elements of oxidative phosphorylation. This then enabled calculation of specific parameters of oxidative phosphorylation including (B) basal respiration, (C) maximal respiration, (D) ATP production and (E) Spare respiratory capacity. Means that do not share the same letter are significantly different (*P* < 0.05)

Subsequently, Seahorse assay technology was utilised to explore the effect of a series of wavelengths on real‐time mitochondrial respiration. Whilst several studies have explored the effect of specific PBM parameters utilising Seahorse technology 31, 32, ours is the first that has explored an array of wavelengths and in particular the use of blue light in PBM. Our data showed that PBM at all wavelengths upregulated both maximal and basal respiratory rates, ATP production and spare respiratory capacity (the amount of extra ATP produced through oxidative phosphorylation available in the case of an increase in energy demand 33) in myotubes (Figure 7, *P* < 0.05), whilst these were only upregulated at a wavelength of 810 nm from myoblasts (*P <* 0.05). Comparatively, previous studies exploring the effects of PBM using a Seahorse analyser only explored the effects of red light (635‐700 nm) and only Chu‐Tan et al found PBM modulated real‐time mitochondrial activity 31. Data from this study suggest that mitochondrial content may influence cellular response to PBM. However, further investigations are required to confirm this finding. Furthermore, our data indicate that blue light promoted greater increases in mitochondrial activity from myotubes compared with near infra‐red (NIR) irradiation. Interestingly, PBM research does not often employ light within the blue range. However, recently, the application of blue light has gathered considerable interest and several authors have provided evidence that blue light not only could be beneficial in reducing inflammation by reducing circulating levels of cytokines 34 and promoting cell proliferation 35. Hence, in future studies, it will be important to explore the response of other cell types to low doses of blue light. However, whilst we have reported blue light induces a greater response compared to NIR light in vitro, it may be wise to consider the possible limitations of blue light in terms of tissue penetration depth. Hence, future studies may aim to evaluate the effects of combining both blue and NIR light to ensure light penetrates target tissue in vivo. The use of blue light may also be considered for superficial injuries including applications for wound healing, which have proven beneficial both in vitro 34 and in vivo 36, 37.

In summary, we demonstrate for the first time that PBM promotes greater increases in mitochondrial respiration in myotubes compared with myoblasts, a cell type with higher levels of mitochondrial content. These data may prove useful in understanding why some patients are more responsive to PBM in vivo as it is well reported there is a great deal of variability in the mitochondrial genome from one individual to the next 38. We also provide novel evidence that blue light could also be effective in promoting mitochondrial respiration. These data provide further evidence supporting the premise that response to PBM in vitro is induced by changes in mitochondrial activity and provide evidence that PBM could be employed to promote increased muscle cell activity. Hence, these data support current findings that indicate the potential effectiveness of PBM in sport performance and rehabilitation following muscle injury 39.

## CONFLICTS OF INTEREST

The authors declare no potential conflicts of interest with respect to the authorship and/or publication of this article.

## AUTHOR BIOGRAPHIES

Please see Supporting Information online.

## Supporting information


**Author Biographies**

**Appendix S1.** Supporting Information
