# Supplementary material for: Differential responses of myoblasts and myotubes to photobiomodulation are associated with mitochondrial number
Source: J Biophotonics. 2019 Feb 20;12(6):e201800411. doi: 10.1002/jbio.201800411 (PMC7065641; doi:10.1002/jbio.201800411)
Supplement: Supplementary file 1 — Author Biographies [file JBIO-12-e201800411-s001.docx]

| 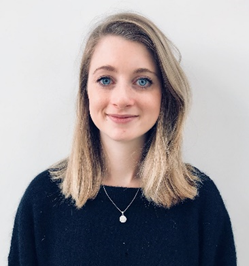 | **Hannah Serrage**- after obtaining her undergraduate degree from Newcastle University, Newcastle upon Tyne, UK in Biomedical Science, she is currently undertaking an iCASE PhD studentship sponsored by Philips. Her research interests involve the evaluation of the effects of Photobiomodulation (PBM) in the management of oral disease in in vitro systems. Her research particularly focuses on dissecting the molecular mechanisms of PBM in disease relevant models through the application of bacteria to in vitro cell cultures. She received the prestigious 3MT award at the IADR 2018 in which she described her research in simple terms in three minutes. |
| --- | --- |
| 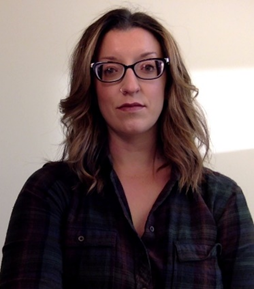 | **Dr Sophie Joanisse** – Sophie completed her PhD in Kinesiology studying the role of muscle stem cells in skeletal muscle remodelling at McMaster University in Hamilton, Canada. She then relocated to the University of Birmingham, UK where she was the Scientific Research Officer overseeing the Mitochondrial Profiling Centre in the School of Sport, Exercise and Rehabilitation Sciences at the University of Birmingham under the supervision of Dr. Andrew Philp. Her research focused on furthering our understanding of mitochondrial biology in various tissues and cell types in response to different interventions. Sophie has since returned to McMaster University where she is a Post-doctoral Research Fellow, her current research focuses on further elucidating the mechanisms underlying the loss of muscle mass and maximizing the efficiency of muscle repair and regeneration in older individuals. |
| 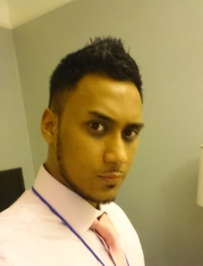 | **Dr Mohammed Hadis** – Research fellow, School of Dentistry, University of Birmingham, Birmingham, UK. His research interests and expertise relate to biomedical applications of lights from photo-curing of composite materials to interaction of light with cells, tissue, molecules and micro-organisms. He provides key skills in the field of photonics for the development of novel technologies and currently working on projects funded by the National Institute of Health Research and The Ministry of Defence. His research has been awarded several prestigious awards including the Paffenbarger Award for outstanding research and the Heraeus Kulzer Award for innovative testing design. |
| 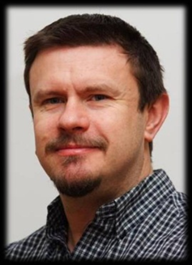 | **Professor Paul Cooper** received his PhD from the University of Birmingham, Birmingham, UK in Cancer Sciences in 1995. He has worked as a post-doctoral researcher at Roswell Park Cancer Institute, New York, US and for Novartis Pharmaceuticals, UK. He conducts research into stem cells, immune/inflammation and tissue regeneration in oral and dental disease and the biomaterials area. He received the prestigious Young Investigator Award from the International Association for Dental Research in 2010. He has >100 full publications and he is currently the Director of Research at the School of Dentistry and leads the Oral Biology teaching. |
| 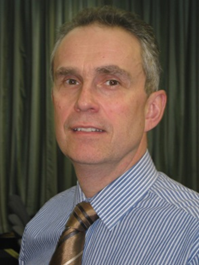 | **Dr Mike Milward** - (Senior Clinical Lecturer / Honorary Consultant in Periodontology, School of Dentistry) Initially trained/qualified as a microbiologist before embarking on undergraduate dental training, following completion he embarked a PhD investigating the role of bacteria in host inflammatory response. His research expertise relates to optimizing approaches for the management of inflammation, infection and tissue repair. He is a calibrated clinical examiner for over 12 clinical trials undertaken over the last 15 years investigating the clinical efficacy of a wide range of oral healthcare products. |
| 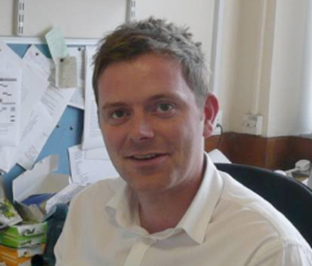 | **Professor William M Palin** - (Professor and Chair in Biomedical Materials Science, University of Birmingham, Birmingham, UK) has an international reputation in the development of novel biomaterials, medical devices and relevant technologies for bone and tooth repair. Significant research fields at the materials science-biological interface include photobiomodulation: the use of light energy to promote accelerated tissue healing, pain-relief and biofunctionalization, and applied biomedical materials science for the design of light curable restorative and therapeutic materials for dental and medical application. Dr Palin has published over 80 papers in peer-reviewed journals and is highly cited, with an h-index of 31. |
| 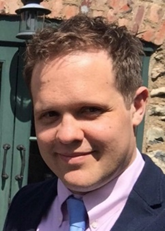 | **Owen Darch** - is a researcher with a PhD in molecular microbiology from the University of Nottingham, Nottingham, UK. He worked for Philips research in Cambridge, UK in the oral healthcare group. There he supported the development of novel Sonicare oral healthcare products, using his background in biofilm research. In addition, he coordinated research projects with several universities, researching basic biology with the aim of developing fundamental technical insights. |
| 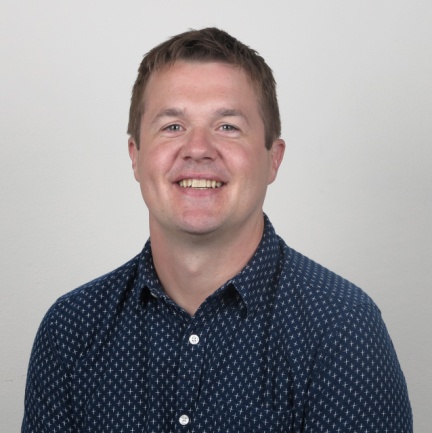 | **Dr Andrew Philp** - is a group leader in the Diabetes and Metabolism Division at the Garvan Institute of Medical Research, where he leads the Mitochondrial Metabolism and Ageing laboratory. Andy completed his PhD in Exercise Physiology at the University of Brighton UK, before completing post-doctoral training at the University of Dundee and the University of California Davis. His group at the Garvan explores the role of mitochondrial metabolism in the progression of muscle deterioration in Diabetes and Ageing, focusing on the therapeutic potential of exercise, pharmacology and nutraceuticals to maintain optimal muscle function across healthspan. Andy has received New Investigator awards from the American Physiological Society, RANK prize nutrition funds and the Biotechnology and Biological Sciences Research Council (BBSRC). Andy is Specialty Chief Editor for Frontiers in Nutrition and serves as an Academic Editor for PLoS One and Translational Sports Medicine. |
