# Supplementary material for: Differential responses of myoblasts and myotubes to photobiomodulation are associated with mitochondrial number
Source: J Biophotonics. 2019 Feb 20;12(6):e201800411. doi: 10.1002/jbio.201800411 (PMC7065641; doi:10.1002/jbio.201800411)
Supplement: Supplementary file 2 — Appendix S1. Supporting Information [file JBIO-12-e201800411-s002.docx]

Supporting Information

***The Differential Responses of Myoblasts and Myotubes to Photobiomodulation correlate with Mitochondrial Number.***

*H. Serrage¹ˑ²ˑ³*, S. Joanisse², P. R. Cooper¹, W. Palin¹, M Hadis¹, O. Darch³, A. Philp²^,4^, M. R. Milward¹*

*School of Dentistry, University of Birmingham, UK ¹*

*School of Sport, Exercise and Rehabilitation Sciences, University of Birmingham, UK²*

*Philips Research, Eindhoven, Netherlands^3^*

*Garvan Institute of Medical Research, Sydney, Australia^4^*


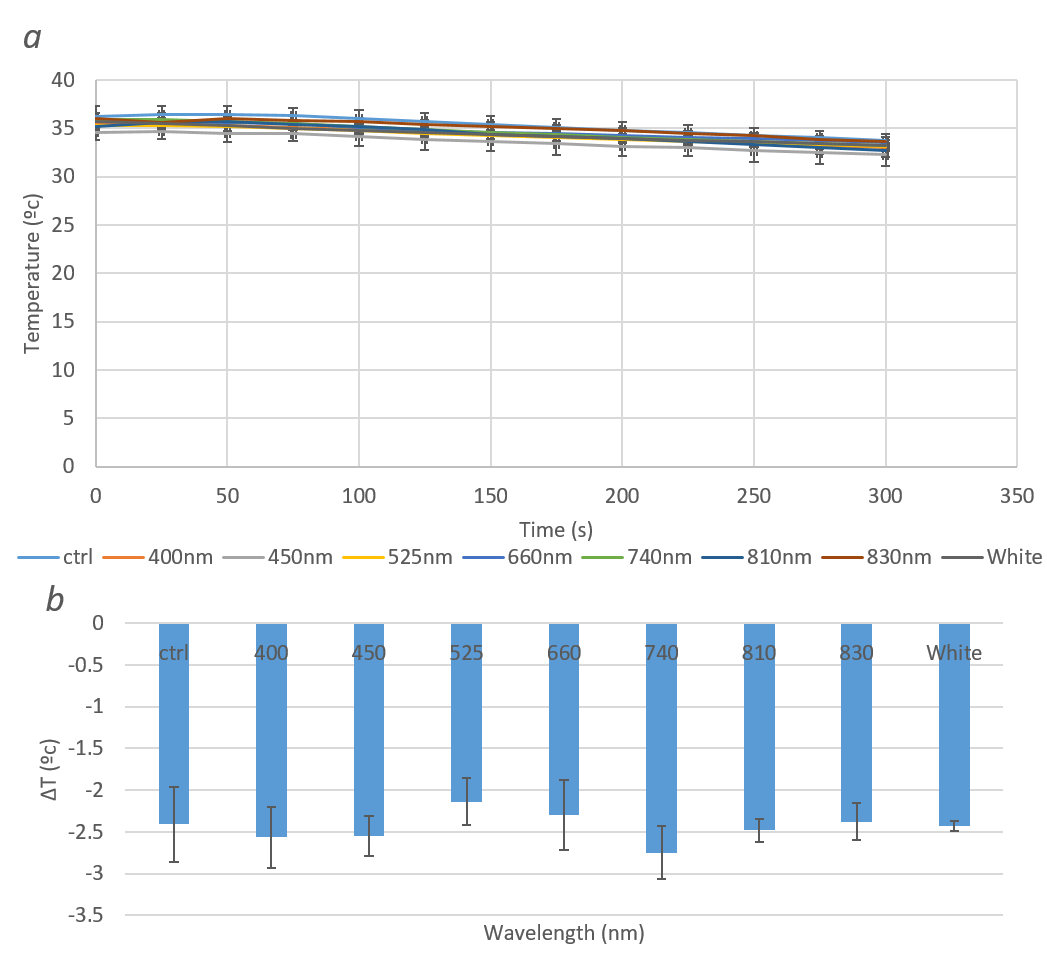


Figure 1: Indicates the effects of PBM on media temperature where a) indicates the average decrease in temperature over a 300s irradiation period and b)shows the average decrease in temperature over a 300s irradiation period across the LED array. The effect of light irradiation on media temperature was assessed to ensure changes in temperature were not exerting a biological affect. Media was aliquoted into a Seahorse microplate and heated to 37ºC . A K-type thermocouple (Maplin, UK; diameter 1.21mm) fixed into a SubMiniature version A (SMA) adapter (6mm outer diameter, 4mm diameter) was placed in media containing wells and irradiated at room temperature (~25ºc) for 240s. Real time measurement of temperature changes were assessed using a multimeter device (Iso-Tech, IDM 207, UK) and logged using data capture software (Virtual DMM, UK).


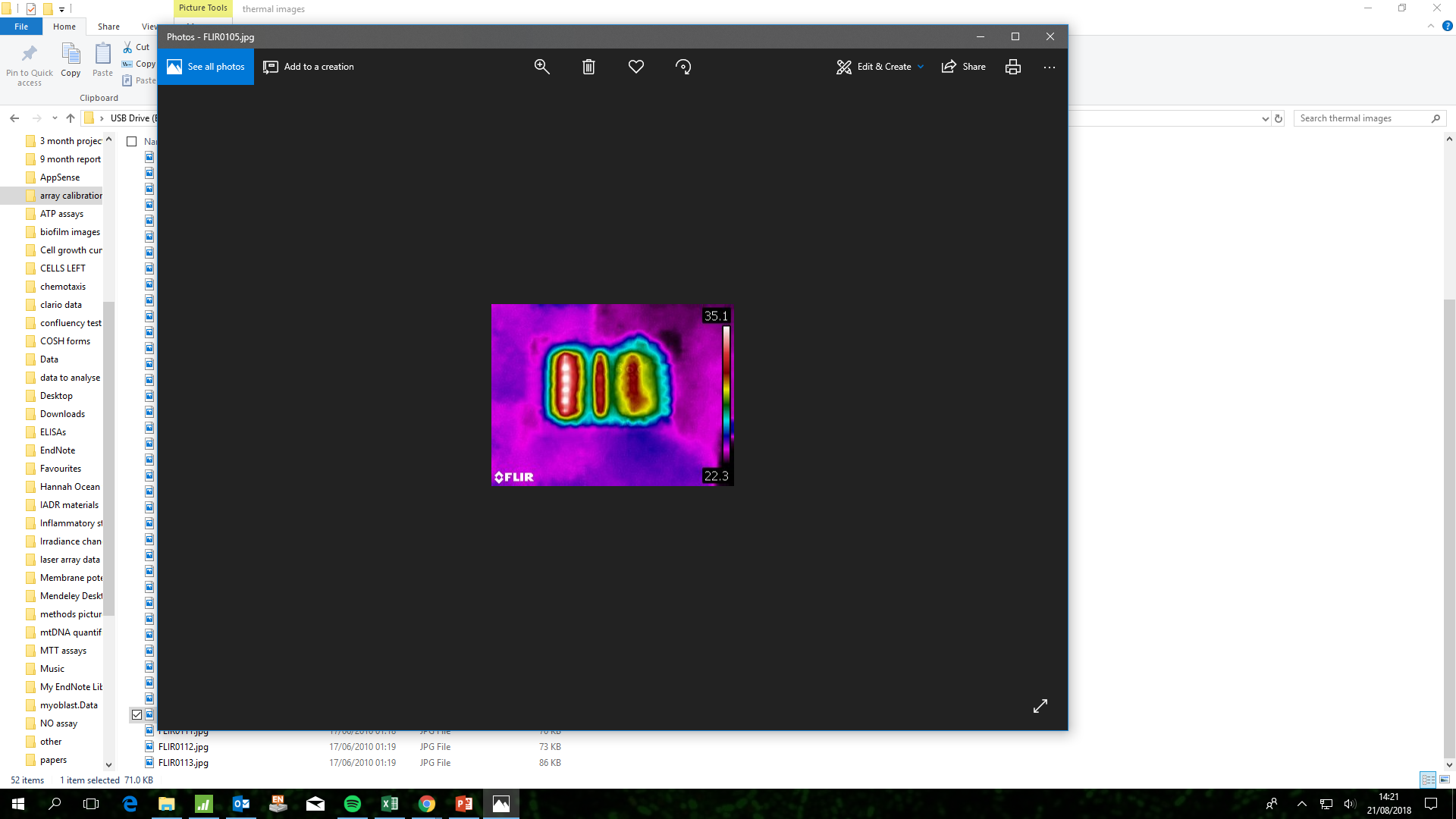

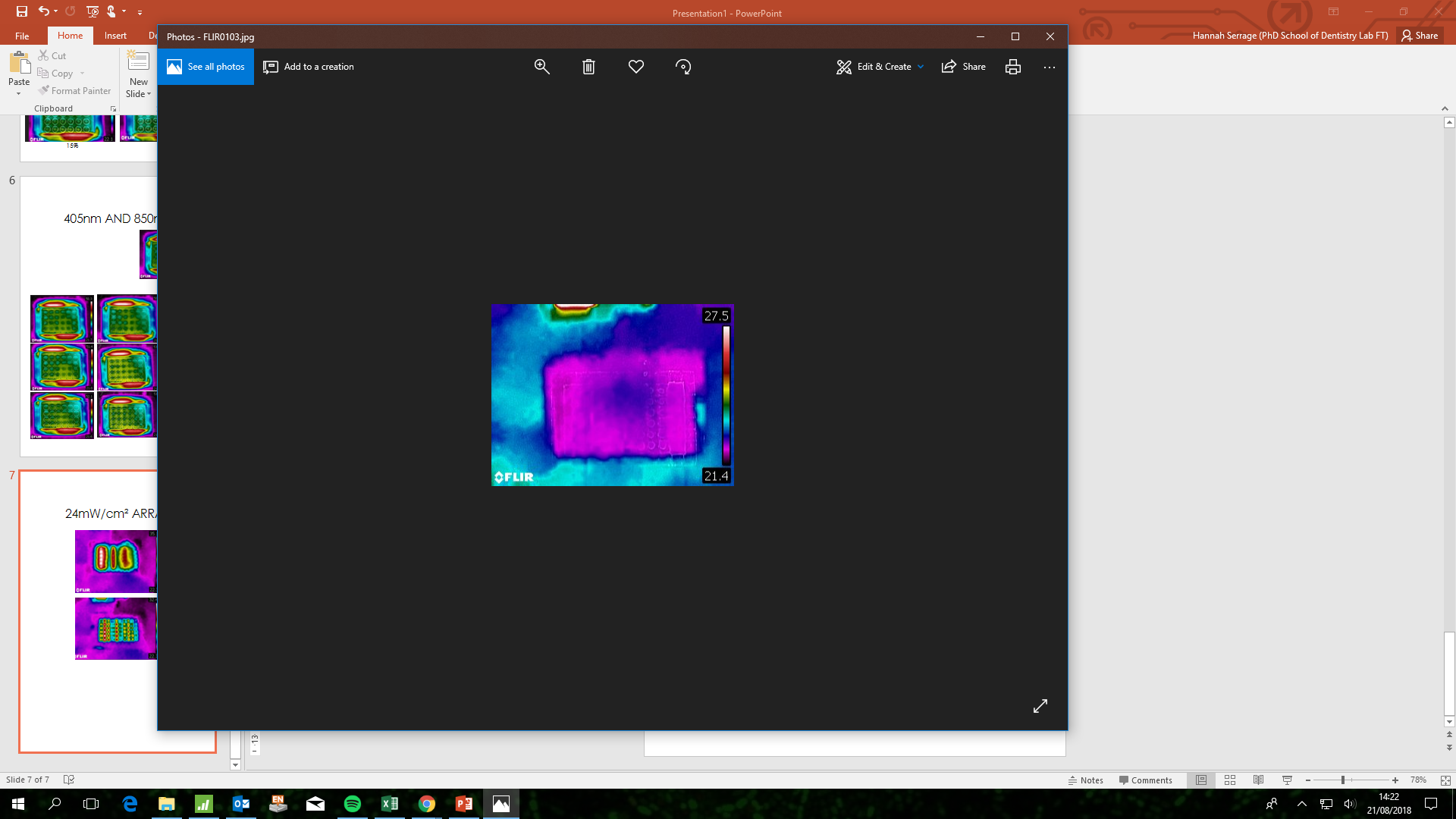


*a*

*b*

Figure 2: Indicates heat map of array when LED array is on where a) shows heat output when all LEDs are switched on without the placement of a plate on top and b) with the Seahorse XFe96 microplate placed on the LED array (24mW/cm²). A thermal image camera (FLIR C2 camera, FLIR, UK) was employed to assess the heat output of LEDs emitting different wavelengths. The camera was positioned 25cm from the array and 3M™ Temflex (3M™, UK) was applied around the exterior of the array (in which no LEDs were located) to reduce emissivity of areas surrounding the irradiated area.


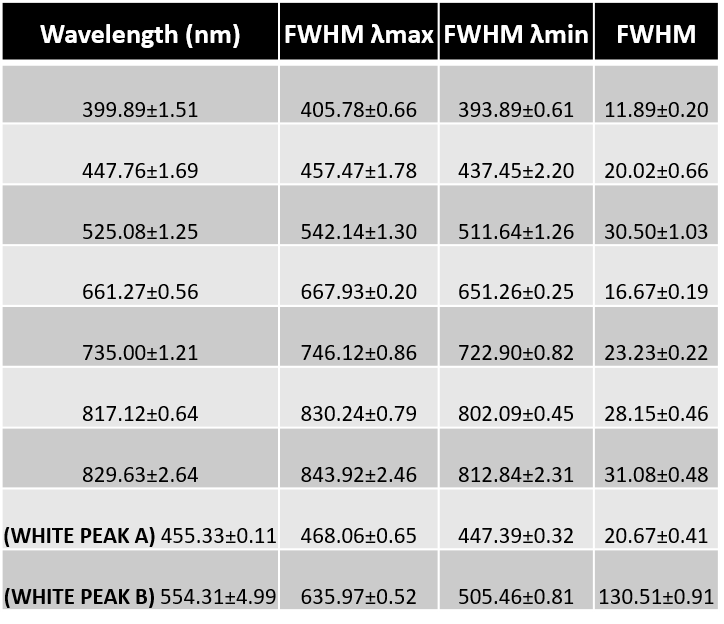


Table 1: Radiometric data calculated from spectral irradiance graphs, confirming accurate and reliable irradiation to cultures in vitro.


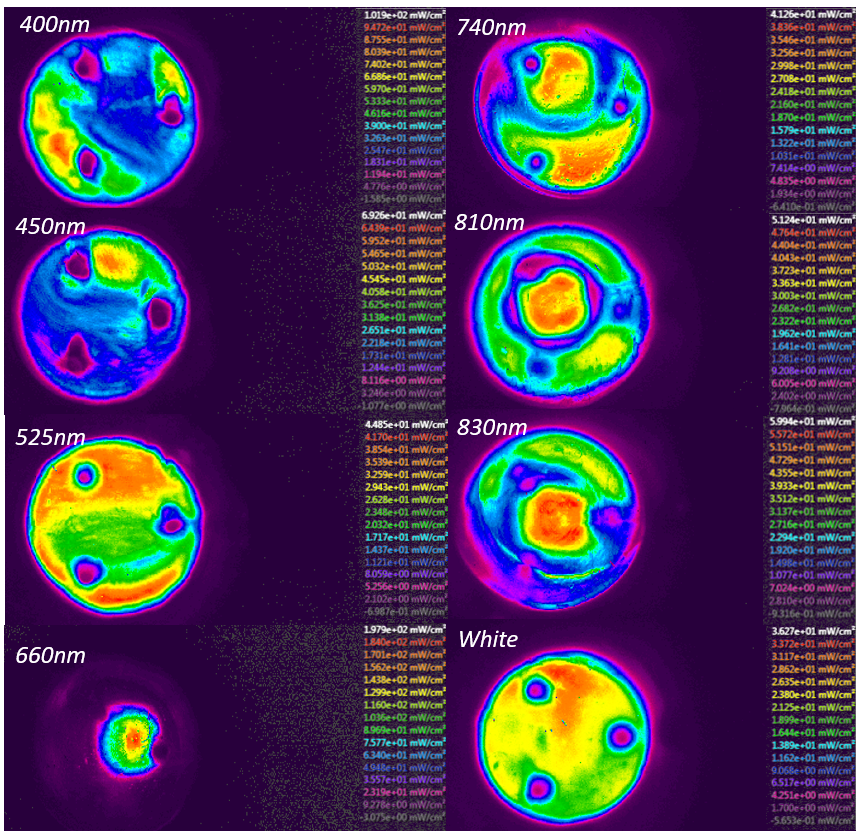


Figure 3: Demonstrates spatial distribution of irradiance of LEDs emitting each wavelength on the array (representative image from each wavelength channel). Images were taken without the presence of a target screen, hence whilst the images seen in figure 3 of the main body of text indicate light delivery in a single plane, in which there is reliable indication of light delivered just to adherent cells at the base of the well, the image above describe the profiles delivered in 3D. Hence, they provide information of the light delivered to the whole well and that emitted outside of the well. Hence, this is not a reliable indication of light delivered to adherent cells and whilst it would have been beneficial to visualise light delivery with the seahorse XFe96 microplate and target screen in place, this was not feasible in the case of this model.


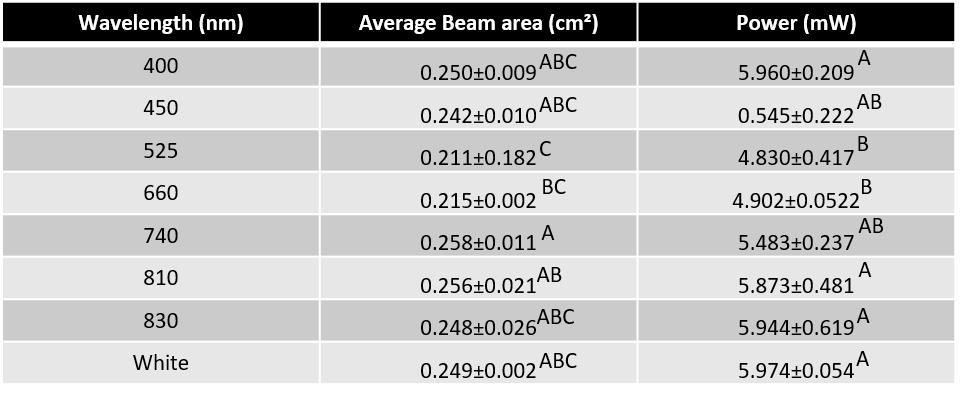


Table 2: indicates differences in average beam area and power output emitted from one wavelength to the next. Means that do not share the same letter are significantly different, in which LEDs emitting wavelengths of 740nm and 810nm (A and AB respectively) exhibit significantly larger beam areas and power outputs than LEDs emitting 525nm light (C, p=<0.05). Average beam area was calculated from diameters provided from use of BeamGage software. Whilst there are difference in average beam area and power output across the array, there were no significant differences in outputs from wavelengths selected for further study (400nm, 450nm and 810nm). Hence, in our model we could rule out the ideology that beam area or power output would influence biological response in vitro.


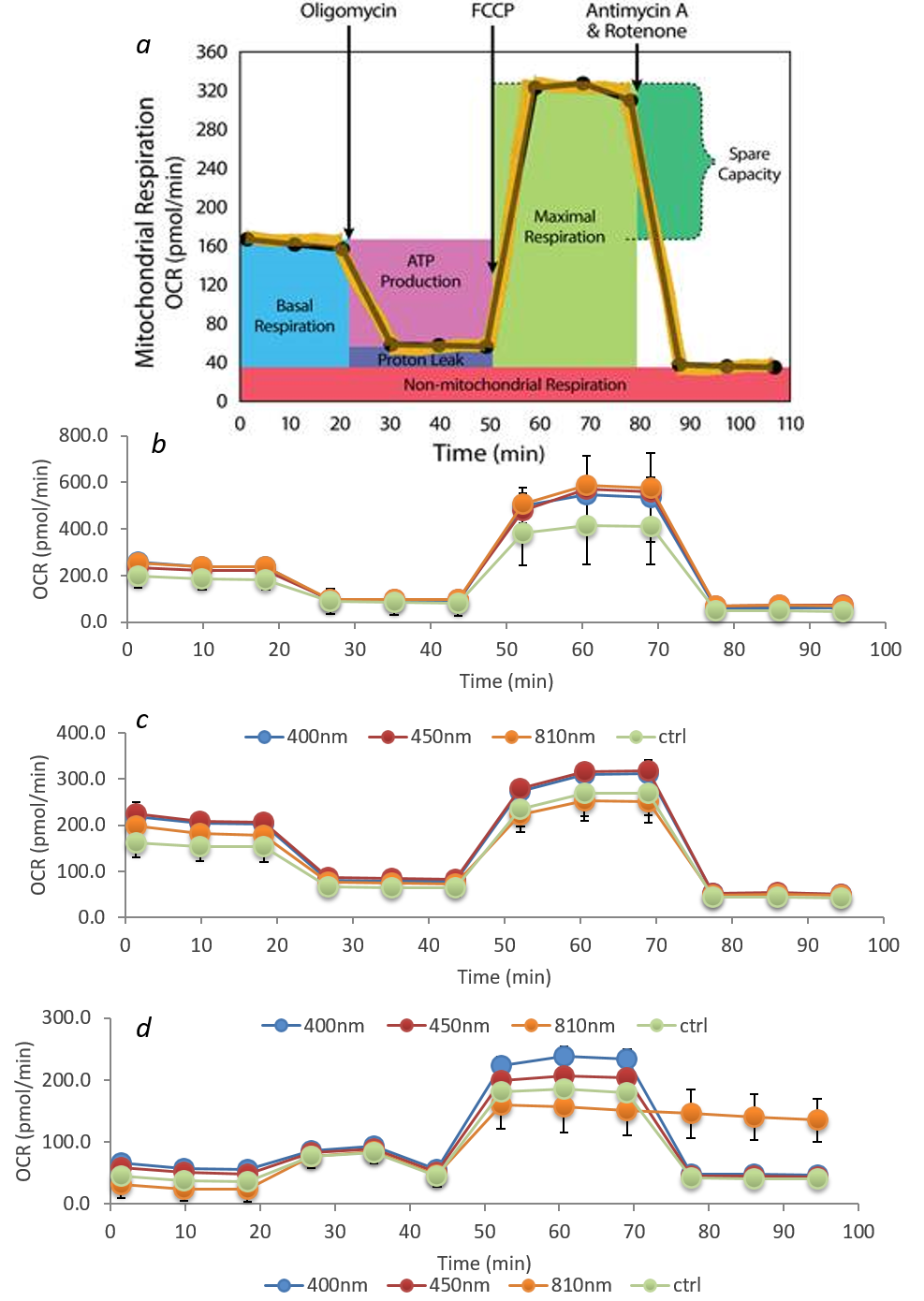


Figure 4: a) Indicates the sequential injection of compounds into the Seahorse system to enable calculation of changes in mitochondrial activity b)Indicates changes in mitochondrial 1hr post-irradiation in which there is overlap in standard deviation and hence no significance c) Indicates changes in mitochondrial activity 8hr post-irradiation where there is much less deviation in outcome and the trend in data is much more similar to the figure depicted by figure 2a d)Indicates changes in mitochondrial activity 24hrs post-irradiation where deviation is again larger and there is an error in the loading of antimycin and rotenone A in 810nm treated wells. Hence, with these data taken into account a time point 8hrs post-irradiation was selected due to reliability. Cells were treated for 30s, 24mW/cm², 0.72J/cm² at wavelengths of 400nm, 450nm or 810nm and evaluated 1,8 and 24hrs post-irradiation. Data here not normalised for protein concentration.

Table 3: Indicates a selection of current literature surrounding the effects of PBM on Myoblasts or Myotubes in which only 54% of articles reported irradiance values and 45% reported beam area. Scopus search involved evaluation of articles containing key words: (‘Photobiomodulation’ OR ‘PBM’ OR ‘Low Level Light Therapy’ OR ‘LLLT’ OR ‘LILT ’OR ‘Photobiostimulation’) AND (‘Myoblast’ OR ‘C2C12’ OR ‘Myotube’).

| Citation | Light Source | Dose | Conclusion |
| --- | --- | --- | --- |
| 1) Mesquita-Ferrari et al, 2015 (1) | **Source:** AlGaAs  **Wavelength:** 780nm  **Power:**  **Frequency:** CW  **Spot area:** 0.04cm² | **Irradiance:** 0.25mW/cm²  **Time:** 20s  **Energy:** 0.2J  **Radiant exposure:** 5J/cm² | Increase in cell viability and creatine kinase activity compared to non-irradiated control. |
| 2) Monici et al, 2013 (2) | **Source:** Multiwave locked system laser  **Wavelength:** 905nm and 808nm  **Power:** 25mW (905nm), 550mW (808nm).  **Frequency:** 909nm, pw 100ns, 808nm cw.  **Spot area:** 5.6cm² | **Irradiance:**  **Time:** 20s x 3  **Energy:** 68J  **Radiant exposure:** | Decrease in cell proliferation with no effect on cell viability. Also increased the production of proteins relevant to cell cycle progress, ATP binding proteins and proteins involved in cytoskeleton rearrangement. |
| 3) Kushibiki et al, 2013 (3) | **Source:** laser  **Wavelength:** 405nm, 664nm and 808nm.  **Power:**  **Frequency:**  **Spot area:** | **Irradiance:** 100mW/cm²  **Time:** 60s or 120s  **Energy:**  **Radiant exposure:** | Blue light (405nm) induced an increase in ROS generation compared to non-irradiated controls. |
| 4) Ferraresi et al, 2015 (4) | **Source:** LEDs  **Wavelength:** 630nm and 850nm  **Power:** 50mW (850nm) and 100mW (630nm) per LED. 1000mW (850nm) and 500mW (630nm) per cluster.  **Frequency:** cw  **Spot area:** 0.2cm² (per LED) and 45cm² (per cluster) | **Irradiance:** 28mW/cm²  **Time:** 90s  **Energy:**  **Radiant exposure:** 2.5J/cm² | Use of both wavelengths synergistically increased the production of ATP and the mitochondrial membrane potential compared to non-irradiated controls. |
| 5) Teuschl et al, 2015 (5) | **Source:** LED  **Wavelength:** 470nm and 630nm  **Power:**  **Frequency:** cw  **Spot area:** | **Irradiance:**  **Time:** 10 mins x 5  **Energy:**  **Radiant exposure:** | 470nm decreased proliferation, augmented apoptosis and increased necrosis rates. 630nm did not alter apoptosis, increased proliferation and decreased rate of wound closure. |
| 6) Lui et al, 2006 (6) | **Source:** LED  **Wavelength:** 640nm  **Power:**  **Frequency:** cw  **Spot area:** | **Irradiance:** 0.229 – 1.67mW/cm²  **Time:** 15 mins x 2  **Energy:**  **Radiant exposure:** | 0.848mW/cm² induced an increase in cell proliferation compared to simvastatin treated cells. |
| 7) Silva et al, 2015 (7) | **Source:** GaAs  **Wavelength:** 685nm and 830nm  **Power:** 35mW (685nm) and 100mW (830nm)  **Frequency:** cw  **Spot area:** | **Irradiance:**  **Time:** 16-56s (685nm) and 6-20s (830nm)  **Energy:**  **Radiant exposure:** 2-7J/cm² | Induced myoblast differentiation to myotubes and the upregulation of MyoD and myogenin. Decreased extracellular production of ATP but increased intracellular production. |
| 8) Nyugen et al, 2013 (8) | **Source:** laser  **Wavelength:** dual output 808nm and 980nm  **Power:** 3W  **Frequency:** cw, 5Hz  **Spot area:** 10.2cm² | **Irradiance:**  **Time:** 53s x 4  **Energy:**  **Radiant exposure:** 22.8J/cm² | Increased production of mitochondrial signalling proteins, oxidative stress and the production of upstream regulatory proteins. |
| 9) Shefer et al, 2008 (9) | **Source:** HeNe laser  **Wavelength:** 632.8nm  **Power:** 4.5mW  **Frequency:** cw  **Spot area:** 1.8mm beam diameter | **Irradiance:** 200mW/cm²  **Time:** 3s  **Energy:**  **Radiant exposure:** 0.6J/cm² | Induction of myoblasts to differentiate into myofibers. |
| 10) Mesquita-Ferrari et al, 2011 (10) | **Source:** GaAlAs and InGaAlP laser  **Wavelength:** 780nm and 660nm  **Power:** 15-70mW (780nm) and 15-40mW (660nm)  **Frequency:** cw  **Spot area:** | **Irradiance:** 37.5 – 175mW/cm² (780nm) and 100mW/cm² (660nm)  **Time:** 10s  **Energy:**  **Radiant exposure:** 3.8 – 17.5J/cm² (780nm) and 3.8 – 10J/cm² (660nm) | LLLT had no effect on cell proliferation 48h and 72h post-irradiation. |
| 11) Xu et al, 2008 (11) | **Source:** GaAlAs laser diode  **Wavelength:** 810nm  **Power:**  **Frequency:** cw  **Spot area:** | **Irradiance:**  **Time:** 10s  **Energy:**  **Radiant exposure:** 0.33 – 14.16J/cm² | Electrical stimulation induced mitochondrial dysfunction was improved by light irradiation between fluency values of 0.33 and 8.22J/cm². |

***References***

1. Mesquita-Ferrari RA, Alves AN, de Oliveira Cardoso V, Artilheiro PP, Bussadori SK, Rocha LA, et al. Low-level laser irradiation modulates cell viability and creatine kinase activity in C2C12 muscle cells during the differentiation process. Lasers in Medical Science. 2015;30(8):2209-13.

2. Monici M, Cialdai F, Romano G, Corsetto PA, Rizzo AM, Caselli A, et al. Effect of IR laser on myoblasts: Prospects of application for counteracting microgravity-induced muscle atrophy. Microgravity Science and Technology. 2013;25(1):35-42.

3. Kushibiki T, Hirasawa T, Okawa S, Ishihara M. Blue laser irradiation generates intracellular reactive oxygen species in various types of cells. Photomedicine and Laser Surgery. 2013;31(3):95-104.

4. Ferraresi C, Kaippert B, Avci P, Huang YY, De Sousa MVP, Bagnato VS, et al. Low-level laser (light) therapy increases mitochondrial membrane potential and ATP synthesis in C2C12 myotubes with a peak response at 3-6 h. Photochemistry and Photobiology. 2015;91(2):411-6.

5. Teuschl A, Balmayor ER, Redl H, Van Griensven M, Dungel P. Phototherapy with LED light modulates healing processes in an in vitro scratch-wound model using 3 different cell types. Dermatologic Surgery. 2015;41(2):261-8.

6. Liu J, Chen XY, Liu CY, Wang SX, Guo H, Xu XY, et al. Photobiomodulation of red light emitting diodes in C2C12 cell proliferation. Chinese Journal of Clinical Rehabilitation. 2006;10(13):107-9.

7. Silva LMG, Da Silva CAA, Da Silva A, Vieira RP, Mesquita-Ferrari RA, Cogo JC, et al. Photobiomodulation protects and promotes differentiation of C2C12 myoblast cells exposed to Snake venom. PLoS ONE. 2016;11(4).

8. Nguyen LMD, Malamo AG, Larkin-Kaiser KA, Borsa PA, Adhihetty PJ. Effect of near-infrared light exposure on mitochondrial signaling in C2C12 muscle cells. Mitochondrion. 2014;14(1):42-8.

9. Shefer G, Ben-Dov N, Halevy O, Oron U. Primary myogenic cells see the light: Improved survival of transplanted myogenic cells following low energy laser irradiation. Lasers in Surgery and Medicine. 2008;40(1):38-45.

10. Mesquita-Ferrari RA, Ribeiro R, Souza NH, Silva CA, Martins MD, Bussadori SK, et al. No effect of low-level lasers on in vitro myoblast culture. Indian journal of experimental biology. 2011;49(6):423-8.

11. Xu X, Zhao X, Liu TCY, Pan H. Low-intensity laser irradiation improves the mitochondrial dysfunction of C2C12 induced by electrical stimulation. Photomedicine and Laser Surgery. 2008;26(3):197-202.
